# Multidisciplinary Expert Guidance for the Management of Severe Bleeding on Oral Anticoagulation: An Algorithm for Practicing Clinicians

**DOI:** 10.1055/a-2464-2887

**Published:** 2024-12-13

**Authors:** Siraj Mithoowani, Tammy Bungard, Lana Castellucci, Mark Crowther, Kerstin de Wit, Dar Dowlatshahi, Nauzer Forbes, Katie Lin, Deborah M. Siegal

**Affiliations:** 1 Department of Medicine, McMaster University, Hamilton, Canada; 2 Department of Medicine, University of Alberta, Edmonton, Canada; 3 Department of Medicine, University of Ottawa, Ottawa, Canada; 4 Inflammation and Chronic Disease Program, Ottawa Hospital Research Institute, Ottawa, Canada; 5 Department of Emergency Medicine, Queen's University, Kingston, Canada; 6 Department of Medicine, University of Calgary, Calgary, Canada

**Keywords:** anticoagulation, bleeding, anticoagulant reversal, warfarin, DOAC, treatment

## Abstract

Bleeding complications associated with oral anticoagulant (OAC) frequently lead to emergency department visits and hospitalization. Short-term all-cause mortality after severe bleeding is substantial ranging from approximately 10% for gastrointestinal bleeding (the most frequent single site) to approximately 50% for intracranial bleeding. A protocol for multidisciplinary approach to bleeding is needed to (i) ensure rapid identification of patients at risk of adverse outcomes, (ii) optimize delivery of supportive measures, (iii) treat the source of bleeding, and (iv) administer anticoagulant reversal or hemostatic therapies judiciously for patients most likely to benefit. We convened a multidisciplinary panel of experts (emergency medicine, gastroenterology, general internal medicine, hematology, neurology, pharmacy, thrombosis) to review the literature and provide practical guidance including a corresponding algorithm for use at the point of care to assist clinicians in the management of patients with acute severe OAC-related bleeding.

## Clinical Cases

**Case 1:** A 57-year-old man with hypertension, dyslipidemia, and a remote deep vein thrombosis (DVT) treated with amlodipine 10 mg daily, rosuvastatin 10 mg daily, and rivaroxaban 20 mg daily (last taken 3 hours ago) presents to hospital with a 1.5-hour history of acute-onset severe headache and right-sided hemiplegia. There was no preceding trauma. His initial Glasgow Coma Scale (GCS) score was 14 but has decreased to 11 on arrival at the emergency department. He is given 10 mg of labetalol for a blood pressure (BP) of 205/100 mm Hg, which then decreases to 165/95 mm Hg. Computed tomography (CT) of head reveals a left basal ganglia intracerebral hemorrhage. A second dose of labetalol is initiated as the patient begins to vomit. Ondansetron is administered while the emergency physician contemplates intubation for airway protection.
**Case 2:** A 68-year-old woman presents to the emergency department with exertional dyspnea and low energy. She reports a 4-day history of melena stools. Medical history includes hypertension, atrial fibrillation (AF) with a CHADS _2_ score of 1, and a lumbosacral disk herniation. Prescribed medications include dabigatran 150 mg twice daily (last dose taken the morning of the emergency department visit) and candesartan/hydrochlorothiazide 16 mg/12.5 mg daily. Over the past 1 to 2 weeks, she was taking ibuprofen multiple times for back pain. On examination, her heart rate is 102 beats per minute and BP is 128/84. Laboratory investigations reveal hemoglobin 83 g/L (decreased from 148 g/L 8 months ago), international normalized ratio (INR) 1.3, and creatinine 100 μmol/L. Electrocardiogram shows sinus tachycardia.
**Case 3:** An 87-year-old man presents to an urgent care center with spontaneous epistaxis for the past 40 minutes. Past medical history includes AF with a CHADS _2_ score of 5 based on age, hypertension, congestive heart failure, and prior stroke. Home medications include apixaban 5 mg twice daily, bisoprolol 2.5 mg twice daily, sacubitril/valsartan 49 mg/51 mg twice daily, and dapagliflozin 10 mg daily. His vital signs are stable, but the bleeding continues despite applying external nasal compression.

## Introduction

More than 40 million prescriptions for oral anticoagulants (OAC) including warfarin and direct oral anticoagulants (DOACs) are written annually in North America. 1 2 Currently available DOACs, their mechanisms of action, pharmacokinetics, and dosing for different indications are shown in Table 1 . The most common indication for OAC use is atrial fibrillation (AF) which is associated with a 5-fold increase in the risk of ischemic stroke and affects over 59 million individuals worldwide. 3 Without OACs, the annual risk of ischemic stroke in AF is approximately 5%; OACs reduce this risk by approximately 65%. 4 Venous thromboembolism (VTE), which includes deep vein thrombosis (DVT) and pulmonary embolism (PE), is the third most common cardiovascular disease affecting about 1 to 2 per 1000 persons per year with mortality rates of 9 to 32 per 100,000. 5 The risk of VTE increases with age with incidence rates of 2 to 10 per 1000 persons per year after 60 years of age. Given an aging population, these conditions, along with their treatment-related complications, are expected to become even greater health problems.

**Table 1:** DOAC mechanism, pharmacokinetics, and dosing

	Dabigatran 118	Apixaban 51	Rivaroxaban 52	Edoxaban 53
**Mechanism**	Direct thrombin inhibitor	Factor Xa inhibitor	Factor Xa inhibitor	Factor Xa inhibitor
**Renal excretion**	80%	25%	35%	50%
** Half-life ^a^**	7–17 hours	8–12 hours	7–11 hours	10–14 hours
**Atrial fibrillation**	150 mg BID 110 mg BID if ≥80 years or if 75 years with a bleeding risk factor	5 mg BID 2.5 mg BID if ≥2 or more of the following: ≥80 years, ≥60 kg, serum creatinine ≥133 umol/L	CrCl >50 mL/min: 20 mg OD with food CrCl 15–50 mL/min 15 mg OD with food	CrCl >50 mL/min: 60 mg daily 30 mg daily if ≥1 of the following: CrCl 15–50 mL/min, ≤60 kg, concomitant P-gp inhibitor
**Venous thromboembolism**	After 5–10 days of parenteral therapy 150 mg BID 110 mg BID if ≥80 years or if ≥75 years with a bleeding risk factor	Acute treatment: 10 mg BID × 7 days then 5 mg BID Secondary prevention: 5 mg BID or 2.5 mg BID	Acute treatment: 15 mg BID × 3 weeks then 20 mg OD with food Secondary prevention: 20 mg OD or 10 mg OD	After 5–10 days of parenteral therapy 60 mg daily 30 mg daily if CrCl 15–50 mL/min, weight ≤60 kg, or taking certain concomitant P-gp inhibitor medications
** Elective orthopedic surgery prophylaxis b**	110 mg (day of surgery), then 220 mg once daily if CrCl >50 mL/min	2.5 mg BID	10 mg OD	Not indicated
**Stable CAD/PAD**	Not indicated	Not indicated	2.5 mg BID with aspirin 75–100 mg OD	Not indicated

Abbreviations: BID, twice daily; CAD, coronary artery disease; CrCl, creatinine clearance; DOAC, direct oral anticoagulant; OD, once daily; PAD, peripheral artery disease.

Notes: ^a^ Assuming CrCl ≥50 mL/min, half-life will be prolonged with renal insufficiency or liver disease.

b Knee replacement, duration of therapy 10–14 days; hip replacement, duration of therapy 28–35 days.

OAC-associated bleeding is the most common adverse drug event that leads to emergency department visits, hospital admissions, and death. 6 7 OAC use is limited by serious (major) bleeding which affects approximately 2 to 4% of OAC-treated patients per year. 8 9 Another 4 to 22% experience clinically relevant non-major (CRNM) bleeding, depending on bleeding definitions, anticoagulant type, duration of treatment, population under study, and study design. 10 11 12 13 14 15 16 17 18 19 20 21 22 23 24 25 26 27 28 29 Outcomes after bleeding vary according to bleed site, and assessment of bleed severity (including fatal bleeding) is limited by a lack of validated standardized definitions. All-cause mortality after major bleeding is substantial ranging from approximately 10% for gastrointestinal bleeding (the most frequent single site) to 50% for intracranial bleeding in contemporary studies of patients treated with OACs. 30 31 32 33 34 35

Patient stabilization and bleeding cessation are priorities when managing severe OAC-related bleeding. A protocolized, multidisciplinary approach to bleeding emergencies ensures (i) rapid identification of patients at risk of adverse outcomes, (ii) optimal delivery of supportive measures, (iii) definitive treatment of bleeding source (as appropriate), and (iv) judicious administration of anticoagulant reversal or hemostatic therapies.

**Fig. 1:**
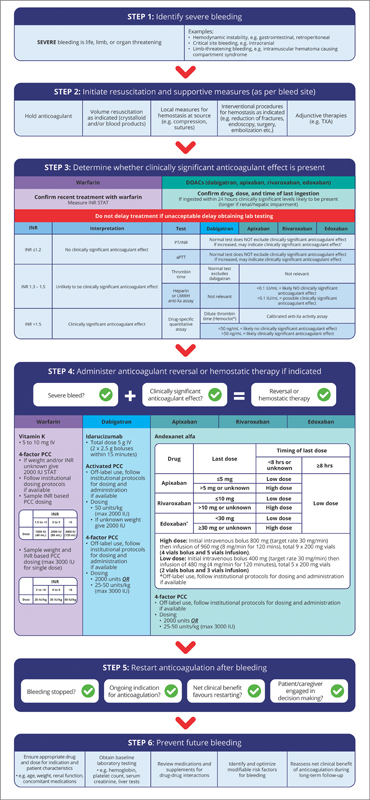
Severe Anticoagulant-Related Bleeding Management Algorithm.

We convened a multidisciplinary panel of experts (emergency medicine, gastroenterology, general internal medicine, hematology, neurology, pharmacy, thrombosis) to review and summarize current evidence and provide practical guidance with a corresponding algorithm ( Fig. 1 ) to assist clinicians managing patients with acute severe OAC-related bleeding at the point of care. The guidance was directly informed by current scientific evidence and clinical expertise in key specialty areas which were collected and discussed during meetings of the author group. In addition to landmark primary studies, we reviewed clinical practice guidelines and consensus statements from the American Society of Hematology (ASH), International Society on Thrombosis and Haemostasis (ISTH), American College of Cardiology (ACC), National Advisory Committee on Blood and Blood Products (NAC), and Thrombosis Canada. 36 37 38 39 We developed representative clinical cases to support the guidance and demonstrate application of the framework in clinical practice.

## Step 1: Identify Severe Bleeding

Bleeding severity is the primary factor influencing the need to interrupt anticoagulation, initiate resuscitation, and proceed with hemostatic or surgical interventions. Although definitions of bleeding used in clinical trials (e.g., ISTH) are widely known, they have important limitations for managing patients with bleeding in clinical practice. By emphasizing laboratory parameters (e.g., hemoglobin concentration) or treatments (e.g., transfusion) without including signs of severe bleeding (i.e., hemodynamic instability), they may be difficult to apply at the onset of a bleeding emergency. 40 Initially, there may be uncertainty about the location of the bleed (e.g., bleeding in the retroperitoneal space) or the patient's response to resuscitation (e.g., intravenous [IV] fluids or packed red blood cell transfusions). Additionally, patient-specific factors including elderly age, frailty, and comorbidities (e.g., chronic kidney or liver disease, or prior life-threatening bleeding) that confer worse prognosis are not captured in clinical trial definitions of OAC-associated bleeding. Therefore, for this algorithm, we adopted a pragmatic definition of “severe” bleeding to align guidance with acute presentations encountered in routine clinical practice for use at the point of care: (i) bleeding causing hemodynamic instability (e.g., hypotension and/or tachycardia requiring treatment with or without vasopressor use), (ii) bleeding involving a critical site (e.g., intracranial or pericardial bleeding), or (iii) limb-threatening bleeding (e.g., intramuscular hematoma causing compartment syndrome).

## Step 2: Initiate Resuscitation and Supportive Measures (As per Bleed Site)

Temporary interruption of anticoagulation, irrespective of thrombotic risk, is warranted for virtually all patients with *severe* anticoagulant-associated bleeding. Initial evaluation should focus on assessing hemodynamic stability and ruling out end-organ dysfunction from bleeding. Vital sign abnormalities associated with moderate or large volume blood loss include resting tachycardia, postural hypotension or dizziness, or a postural pulse increment of >30 beats per minute. 41 Other signs of hemorrhagic shock include altered mentation and decreased urinary output. Patients with hemodynamic instability require urgent resuscitation; typical measures include insertion of large-bore peripheral IV line(s), administration of IV fluid, and transfusion with packed red cells, if necessary. Other blood products, including frozen plasma, platelets, and cryoprecipitate or fibrinogen concentrate, may also be indicated as part of a massive transfusion protocol. Urgent consultation with a specialist to assist with hemostatic interventions (e.g., endoscopy, fracture reduction, embolization, surgery) may be necessary depending on the bleed site.

### Tranexamic Acid

Tranexamic acid (TXA) has benefit in patients with or at risk of bleeding (e.g., trauma, postpartum hemorrhage, orthopedic surgery, cardiac surgery) and may be considered despite a lack of data in patients with OAC-related bleeding. The usual dose of TXA is 1 to 1.5 g orally every 8 to 12 hours, or 10 mg/kg IV every 8 hours with dose reductions for renal impairment. A systematic review of 22 studies enrolling 49,538 patients with nonsurgical bleeding showed that TXA did not increase the risk of stroke (relative risk [RR] 1.10; 95% confidence interval [CI] 0.68–1.78), myocardial infarction (RR 0.88; 95% CI 0.43–1.84), PE (RR 0.97; 95% CI 0.75–1.26), or DVT (RR 0.99; 95% CI 0.70–1.41) compared with placebo or no TXA. 42

In the HALT-IT trial, patients with severe gastrointestinal bleeding (only 9% on anticoagulants) treated with IV TXA (1 g over 10 minutes followed by 3 g over 24 hours) had a similar risk of death due to bleeding within 5 days of randomization (4% vs. 4%; RR 0.99; 95% CI 0.82–1.18) and a higher risk of VTE (0.8% vs. 0.4%; RR 1.85; 95% CI 1.15–2.98) compared with those treated with placebo. 43 Although these findings suggest caution, it should be noted that the dose of TXA was relatively high compared with other clinical trials.

No large high-quality clinical trials have effectively evaluated the utility of TXA in a specifically anticoagulated patient population. The CRASH-2 trial enrolled 20,207 trauma patients with or at risk of significant hemorrhage and showed that TXA (1 g IV over 10 minutes then 1 g over 8 hours) reduced the risk of death by 1.5% at 4 weeks compared with placebo (14.5% vs. 16.0%; RR 0.91; 95% CI 0.85–0.97). 44 For isolated traumatic brain injury, the CRASH-3 trial showed that TXA administered within 3 hours of injury did not reduce the risk of head injury–related death at 28 days compared with placebo (18.5% vs. 19.8%; RR 0.94; 95% CI 0.86–1.02). 45 A reduction in the risk of head injury–related death was seen in pre-specified analyses that excluded patients with a Glasgow Coma Scale (GCS) score of 3 or bilateral unreactive pupils at baseline (12.5% vs. 14.0%; RR 0.89; 95% CI 0.80–1.01) and in patients with mild-to-moderate head injury (RR 0.78; 95% CI 0.64–0.95) but not in those with severe head injury (0.99 [95% CI 0.91–1.07]). Finally, for spontaneous intracerebral hemorrhage, the TICH-2 trial showed that TXA did not improve functional neurologic status (adjusted odds ratio [aOR] 0.88, 95% CI 0.76–1.0) or mortality (250/1161 [22%] vs. 249/1164 [21%]; adjusted hazard ratio 0.92, 95% CI 0.77–1.10) compared with placebo at 90 days despite early reductions in hematoma growth (at day 2) and death (at day 7). 46 Although the TICH-NOAC trial focused specifically on TXA use in patients on DOACs, it also failed to demonstrate any significant difference in symptomatic hematoma expansion at 24-hour follow-up (38% vs. 45% in TXA vs. placebo groups respectively; OR 0.63, 95% CI 0.22–1.82, *p* = 0.4); the trial was stopped early when only 67 patients enrolled due to exhausted funding, and thus the study was underpowered for its outcome of interest. 47 Ultimately, the results of these combined studies should be interpreted with caution as they did not specifically enroll anticoagulated patients (CRASH-3), excluded anticoagulated patients altogether (TICH-2), or were underpowered for the outcome of interest (TICH-NOAC).

Unlike TXA, off-label use of recombinant factor VIIa (rFVIIa) for OAC-related bleeding is generally avoided in favor of prothrombin complex concentrate (PCC) or specific DOAC reversal drugs (andexanet alfa or idarucizumab), which are discussed further below. Recombinant FVIIa is unlikely to inhibit the anticoagulant effect of factor Xa inhibitors and is associated with an increased risk of thrombosis when prescribed off-label in individuals without hemophilia. 48 49 50

## Step 3: Determine Whether Clinically Significant Anticoagulant Effect is Present

Ascertaining the time of last OAC ingestion is key for determining the likelihood of clinically significant anticoagulant effect to guide the judicious use of reversal or hemostatic therapies for patients most likely to benefit. For DOACs, clinically significant anticoagulant effect is likely present if ingested within the previous 24 hours (about 2 to 3 half-lives) or longer in patients with renal/hepatic impairment ( Table 1 ).

In practice, it may not be feasible to ascertain OAC levels within an acceptable timeframe to guide initial management during emergencies, particularly for DOACs. Coagulation tests such as the international normalized ratio (INR), prothrombin time (PT), and activated thromboplastin time (aPTT) are routinely measured in patients with bleeding emergencies but are not generally accurate or reliable for detecting clinically significant DOAC levels primarily due to poor sensitivity, although this varies depending on the assay used. The effect of DOACs on laboratory tests is summarized in Table 2 . An exception is the thrombin time (TT) which is very sensitive to the presence of even small amounts of dabigatran, such that a normal TT excludes a clinically important dabigatran level. Calibrated DOAC-specific assays (e.g., dilute thrombin time or ecarin clotting time for dabigatran and drug-specific anti-Xa activity assays for factor Xa inhibitors) can reliably quantify DOAC concentration, but they are not routinely available and may have limited clinical utility in emergencies because of long turnaround time for results relative to clinical urgency. Anti-Xa heparin assays can detect the presence of factor Xa inhibitors, but may not be sufficiently sensitive to rule out clinically significant levels and are also not widely available. There is an urgent need to develop and evaluate validated assays that can reliably identify patients with clinically significant DOAC drug levels rapidly at the point of care to determine which patients are most likely to benefit from reversal or hemostatic therapies.

**Table 2:** Guide to interpreting laboratory testing in the presence of DOACs

Test	Dabigatran	Apixaban	Rivaroxaban	Edoxaban
PT/INR	Normal test does NOT exclude clinically significant anticoagulant effect If increased, may indicate clinically significant anticoagulant effect a			
aPTT	Normal test does NOT exclude clinically significant anticoagulant effect If increased, may indicate clinically significant anticoagulant effect a			
Thrombin time	Normal test excludes dabigatran	Not relevant		
Heparin or LMWH anti-Xa assay	Not relevant	< 0.1 IU/mL = likely NO clinically significant anticoagulant effect > 0.1 IU/mL = possible clinically significant anticoagulant effect		
Drug-specific quantitative assay	Dilute thrombin time (Hemoclot)	Calibrated anti-Xa activity assay		
	<50 ng/mL = likely no clinically significant anticoagulant effect b ≥50 ng/mL = likely clinically significant anticoagulant effect b			

Abbreviations: aPTT, activated partial thromboplastin time; DOACs, direct oral anticoagulants; INR, international normalized ratio; LMWH, low-molecular-weight heparin; PT, prothrombin time; TT, thrombin time.

Notes: The term “clinically significant” anticoagulant effect refers to levels that may contribute to bleeding. The threshold for clinically significant anticoagulant effect is unknown.

a Suggests clinically significant anticoagulant effect in the absence of another cause of coagulopathy (e.g., massive transfusion, disseminated intravascular coagulopathy, coagulopathy of liver disease, vitamin K deficiency, warfarin, a coagulation factor inhibitor, factor deficiency).

b The level chosen (<50 ng/mL) is extrapolated from observations in clinical trials and is consistent with other guidelines. 37

### Factor Xa Inhibitors (Apixaban, Rivaroxaban, Edoxaban)

The half-life of factor Xa inhibitors in older adults is around 10 hours but they undergo renal and hepatic clearance with possible prolonged half-life in the setting of renal or hepatic impairment ( Table 1 ). 51 52 53 Factor Xa inhibitors can prolong the PT and/or aPTT, and increase the INR to a variable degree depending on the specific drug, laboratory reagent, and coagulation instrument used for testing. 54 55 A detailed discussion is beyond the scope of this article. Briefly, elevations in INR and/or prolongation of aPTT may indicate the presence of clinically significant factor Xa inhibitor levels, but these tests have low sensitivity and *cannot reliably rule out* clinically significant levels of factor Xa inhibitor. In addition, INR and aPTT may be abnormal for reasons unrelated to OAC exposure particularly among critically ill patients, including disseminated intravascular coagulation (DIC), liver disease, massive transfusion, vitamin K deficiency, or pre-analytic laboratory error (i.e., low specificity), and should be interpreted with caution.

Drug-specific chromogenic anti-Xa assays can reliably quantify factor Xa inhibitor drug levels, but they are not routinely available in many hospital laboratories. 54 55 Laboratory testing is further complicated by a lack of established therapeutic levels, uncertainty about the relationship between DOAC drug levels measured and the extent of hemostatic impairment, and its impact on bleeding. Despite these limitations, if testing is locally available and reported within an acceptable timeframe, drug level determination is reasonable at initial presentation to help guide initial management and/or complications (e.g., refractory bleeding, surgery). Unfractionated heparin (UFH) or low-molecular-weight heparin (LMWH) calibrated anti-Xa assays are more widely available than drug-specific assays and are highly sensitive to the presence of factor Xa inhibitors, but anti-Xa thresholds to identify patients with clinically significant drug levels (>50 ng/mL) vary widely between commercially available assays limiting their practical utility in the absence of local laboratory validation. 56 57 An undetectable or very low UFH or LMWH anti-Xa level (<0.1 IU/mL) suggests that there is likely no clinically significant circulating anticoagulant, but the precise threshold to rule out anticoagulant exposure remains uncertain and varies between assays.

## Step 4: Administer Anticoagulant Reversal or Hemostatic Therapy if Indicated

Anticoagulant reversal or hemostatic therapy is indicated for patients with severe bleeding, in whom clinically significant anticoagulant effect is suspected (i.e., as per time of last dose and renal function for DOACs as per above) or confirmed.

### Warfarin

Urgent warfarin reversal involves restoring the production of vitamin K-dependent coagulation factors with IV vitamin K and rapid supplementation of vitamin K–dependent coagulation factors with four-factor prothrombin complex concentrate (4F-PCC) (preferred) or plasma (if 4F-PCC is unavailable) until production of coagulation factors is restored. 58 59 60 In a randomized trial of 4F-PCC versus plasma for warfarin-associated major bleeding, a higher proportion of patients treated with 4F-PCC achieved rapid reduction of INR (62% vs. 10%) compared with plasma. Rates of effective hemostasis (72% vs. 65%) and thrombotic events (8% vs. 6%) were similar between groups. 61 4F-PCC is recommended by all major guideline panels for the reversal of warfarin in patients with severe bleeding. 38 39 62 63

Vitamin K directly counteracts the anticoagulant effect of warfarin. IV administration is preferred for emergencies as the speed of coagulation factor production is faster (12 to 14 hours) than that of oral administration (24 to 48 hours). Dosing protocols vary and should align with institutional protocols and product monographs. 4F-PCC is typically administered at a dose of 1000 to 3000 international units (IU) IV depending on the degree of INR elevation and body weight (example in Table 3 ). If the INR or weight are unknown, then a fixed dose of 2000 IU can be administered empirically for severe bleeding when delay is unacceptable. PCC is contraindicated for patients with heparin-induced thrombocytopenia (HIT) and is associated with a 1 to 3% risk of thrombosis when used for warfarin reversal. 58 64 65

**Table 3:** Dosing of four-factor prothrombin complex concentrate for warfarin reversal

	Sample INR based dosing			Sample weight and INR based dosing*		
	INR			INR		
	1.5 to <3	3 to 5	>5	2 to <4	4 to 6	>6
**Dose**	1000 IU (40 mL)	2000 IU (80 mL)	3000 IU (120 mL)	25 IU/kg	35 IU/kg	50 IU/kg

Abbreviations: INR, international normalized ratio; IU, international units.

Note: *Single doses should not exceed 3000 IU.

Source: Adapted from National Advisory Committee on Blood and Blood Products. 39

### Dabigatran

#### Specific Reversal of Dabigatran

Idarucizumab is a monoclonal antibody fragment that binds dabigatran with high affinity and reverses its anticoagulant effect ( Table 4 ). 66 67 RE-VERSE AD was a single-arm trial in which dabigatran-treated patients presenting with acute life-threatening bleeding or needing urgent surgery or invasive procedures were treated with idarucizumab (5 g IV). 68 After idarucizumab treatment, dilute thrombin time and/or ecarin clotting time normalized rapidly (an effect that was maintained for up to 24 hours), unbound (active) dabigatran levels decreased, and 68% of participants achieved hemostasis within 24 hours according to pre-specified criteria. At 90 days, thrombotic events occurred in 6% of participants and 73% of those treated for bleeding had resumed antithrombotic therapy.

**Table 4:** Dosing of specific reversal drugs and hemostatic therapies for DOAC-associated bleeding

Dabigatran	Apixaban	Rivaroxaban	Edoxaban
**Idarucizumab** 119 • Total dose 5 g IV (2 × 2.5 g boluses within 15 minutes) **Activated PCC** • Off-label use, follow institutional protocols for dosing and administration if available • Dosing • 50 units/kg (max 2000 IU) • If unknown weight give 2000 IU **4F-PCC** • Off-label use, follow institutional protocols for dosing and administration if available • Dosing • 2000 units OR • 25–50 units/kg (max 3000 IU)	**Andexanet alfa** 120 ** High dose** : Initial intravenous bolus 800 mg (target rate 30 mg/min) then infusion of 960 mg (8 mg/min for 120 minutes), total 9 × 200 mg vials ( **4 vials bolus and 5 vials infusion** ). ** Low dose** : Initial intravenous bolus 400 mg (target rate 30 mg/min) then infusion of 480 mg (4 mg/min for 120 minutes), total 5 × 200 mg vials ( **2 vials bolus and 3 vials infusion** ). *Off-label use, follow institutional protocols for dosing and administration if available **4F-PCC** • Off-label use, follow institutional protocols for dosing and administration if available • Dosing • 2000 units OR • 25–50 units/kg (max 3000 IU)		

Abbreviations: 4F-PCC, four-factor prothrombin complex concentrate; DOAC, direct oral anticoagulant.

**Table:** 

**Drug**	**Last dose**	**Timing of last dose**	
		**<8 hrs or unknown**	**≥8 hrs**
Apixaban	≤5 mg	Low dose	Low dose
	>5 mg or unknown	High dose	
Rivaroxaban	≤10 mg	Low dose	
	>10 mg or unknown	High dose	
Edoxaban*	≤30 mg	Low dose	
	>30 mg or unknown	High dose	

#### Coagulation Factor Concentrates

Data supporting the use of PCC or activated PCC for dabigatran-related bleeding are limited and conflicting, stemming from animal studies, in vitro studies, and small single-arm cohort studies. 69 Consequently, these agents are generally reserved for cases of severe dabigatran-related bleeding when idarucizumab is unavailable.

#### Other Reversal Strategies

Hemodialysis can reduce dabigatran serum concentration, but its use is limited by logistical challenges in initiating dialysis and treatment delays during acute severe bleeding. 70 Although not the primary choice for reversal, it may be considered as an adjunctive treatment for severe or refractory bleeding associated with reduced dabigatran clearance (e.g., acute kidney injury).

### Factor Xa Inhibitors (Rivaroxaban, Apixaban, Edoxaban)

#### Specific Reversal of Factor Xa Inhibitors

Andexanet alfa is a synthetic, inactive factor Xa molecule designed as a decoy to specifically counteract the anticoagulant effects of factor Xa inhibitors ( Table 4 ). 71 It is approved in many countries for the management of major bleeding in patients receiving apixaban or rivaroxaban. Andexanet rapidly reduces anti-Xa activity, the measure of anticoagulant effect, and enhances endogenous thrombin potential in individuals receiving factor Xa inhibitors. 72 The ANNEXA-4 study was a single-arm prospective registration study that assessed andexanet alfa for acute major bleeding events within 18 hours of taking apixaban, rivaroxaban, edoxaban, or enoxaparin. Andexanet was administered via a short infusion (15 to 30 minutes) followed by a 2-hour infusion, with the dosage based on the type and dose of factor Xa inhibitor and time since the last ingestion. 30 Following andexanet treatment, there was >90% reduction in anti-factor Xa activity and 82% of the participants showed excellent or good hemostasis within 12 hours (as evaluated using pre-specified bleed site–specific criteria by an independent adjudication committee) and 10% experienced thromboembolism within 30 days. 73 Like the RE-VERSE AD study, a notable limitation is the absence of a control group.

In the ANNEXA-I trial, individuals experiencing acute intracranial hemorrhage within 6 hours of symptom onset and 15 hours after their last dose of apixaban, edoxaban, or rivaroxaban were randomly assigned to andexanet or usual care (PCC included in 86%). 74 On stopping the trial early for efficacy based on a pre-planned interim analysis, a larger percentage of patients treated with andexanet had excellent or good hemostatic efficacy (63.9% vs. 52.4%), and fewer experienced hematoma increase of ≥12.5 mL (11.6% vs. 19.0%). At 30 days, the rates of mortality (27.8% vs. 25.5%) and favorable functional status among survivors (modified Rankin Scale score ≤3; 28.0% vs. 30.9%) were not significantly different. Thromboembolic events occurred more often in the andexanet group (10.3% vs. 5.6%), particularly ischemic stroke (6.5% vs. 1.5%). Notable methodological limitations include the open-label design, lack of standardized treatment within the usual care group could bias co-intervention use, and short duration of follow-up for assessment of functional status improvements which are generally evaluated at 6 or 12 months to coincide with expected timeframe for hematoma resolution. Consistent with existing literature regarding high-risk bleed characteristics after OAC-related intracerebral hemorrhage, sub-analyses of ANNEXA-I suggest that andexanet may have greater net benefit for hematoma expansion among patients who present earlier from symptom onset, have rapidly expanding hematomas, and have no history of thromboembolic events but additional data are needed. 75 76 77 78 79

In observational studies of patients with bleeding managed in routine clinical practice, those treated with andexanet may experience lower mortality during hospitalization and within 30 days of bleeding compared with those treated with 4F-PCC. 80 81 However, these nonrandomized comparative studies are limited by the likelihood of selection bias, retrospective design, and inadequate adjustment for patient baseline characteristics that affect prognosis.

### Nonspecific Hemostatic Therapies

4F-PCC is used to manage acute severe bleeding in the absence of specific reversal agents for factor Xa inhibitors. Although 4F-PCC does not reverse anticoagulant effect as measured by factor Xa activity, it may aid hemostasis by overcoming anticoagulant effect to support thrombin generation. 82 The effect of 4F-PCC on laboratory indices varies depending on the assay type, the factor Xa inhibitor, and the plasma DOAC concentration. 69 83 The optimal dose of 4F-PCC for bleeding related to factor Xa inhibitors is uncertain, with fixed doses of 1500 to 2000 units or weight-based doses of 25 to 50 units/kg reported ( Table 4 ). 84 In single-arm observational studies conducted in routine clinical practice, up to 85% of patients treated with 4F-PCC for major bleeding on factor Xa inhibitors achieved hemostasis as defined primarily by modified criteria from the ISTH and up to 8% experienced thrombotic events. 33 34 35 In a recent population-based, propensity score–weighted retrospective cohort study of patients with DOAC-related intracranial hemorrhage, neurological recovery (aOR 0.62; 95% CI 0.33–1.16), mortality at 90 days (aOR 1.03; 95% CI 0.70–1.53; *p* = 0.88), in-hospital mortality (aOR 1.11; 95% CI 0.69–1.79; *p* = 0.66), and reduced hematoma expansion (aOR 0.94; 95% CI 0.38–2.31; *p* = 0.90) were similar between patients who received 4F-PCC and those who were managed conservatively without hemostatic therapy. 85

### Considerations for Use of Andexanet or 4F-PCC

The choice between andexanet or 4F-PCC is individualized based on bleed severity and underlying thromboembolic risk ideally using standardized institutional protocols adapted for local context. In the only randomized trial against usual care (86% PCC use), andexanet was more effective at restoring hemostasis in patients with intracranial hemorrhage which is supported by its effects on anti-Xa activity and thrombin generation. However, the risk of arterial thrombotic events, including myocardial infarction and ischemic stroke, was increased and there was no improvement in mortality or functional status, highlighting equipoise regarding its broad use. Andexanet may be favored among individuals with life-threatening bleeding who have no history of thromboembolic events. For patients with intracranial hemorrhage, the presence of high hematoma expansion rates and earlier presentations may favor andexanet use based on preliminary data. 75

## Step 5: Restart Anticoagulation after Bleeding

Patients experiencing severe bleeding are at risk of thromboembolic complications, influenced by factors such as underlying prothrombotic conditions, activation of hemostasis, withdrawal of anticoagulation, administration of reversal and/or hemostatic therapies, surgery, and hospitalization. Thrombotic events are observed in 4 to 10% of patients within 30 days following DOAC-related bleeding. 33 34 35 68 73 Comparable rates are noted in patients treated with 4F-PCC or plasma for warfarin-related bleeding (6 to 8%). 61 Restarting antithrombotic therapy mitigates thromboembolic risk in this setting. For example, the ANNEXA-4 study found no thromboembolic events among participants who resumed antithrombotic therapy, underscoring the importance of evaluating anticoagulation post bleeding cessation. 86 In a population cohort study of adults ≥65 years of age hospitalized for anticoagulant-related bleeding, those who resumed anticoagulation 3 months after the index bleed had a lower thrombosis rate (hazard ratio [HR] 0.62; 95% CI 0.50–0.72) but higher bleeding rate (HR 1.88; 95% CI 1.64–2.17) over the subsequent year. 87

Decisions on restarting anticoagulation after bleeding are challenging due to limited data, the majority of which are derived from warfarin-treated cohorts. The optimal timing for resuming anticoagulation remains unknown. Anticoagulation is permanently discontinued in up to 50% of patients with gastrointestinal bleeding and 70% with intracranial bleeding. 87 88 89 90 91

There is no universal anticoagulation resumption strategy that applies to all bleeds or patients, highlighting the importance of eliciting and understanding patient/caregiver preferences, and seeking multidisciplinary input. The decision to resume anticoagulation involves confirming an indication for ongoing anticoagulation (type of and clinical consequences of thrombosis), weighing the benefits and harms based on rebleeding and thrombosis risks, and considering the site of bleeding (and the availability of definitive treatments). Some conditions for which ongoing OAC therapy should be re-evaluated include: (i) non-valvular AF with low CHADS _2_ score of 0, (ii) first-time provoked VTE more than 3 months ago, (iii) bioprosthetic heart valve without AF >3 months ago, or (iv) a temporary indication for OAC (e.g., post-surgical VTE prophylaxis). 38

In general, for conditions associated with a very high thrombotic risk (>10%/year risk of VTE or arterial thromboembolism), the balance of rebleeding and thrombosis may favor early re-initiation of OAC, recognizing that rebleeding results in prolonged OAC interruption that may increase thrombotic risk further. These conditions include, but are not limited to, mechanical heart valve, high-risk AF (e.g., valvular AF, high CHADS _2_ score of ≥5, or AF associated with an ischemic stroke/transient ischemic attack [TIA] within 3 months), VTE within 3 months, intra-cardiac thrombosis, or a prior thromboembolic event associated with interruption of anticoagulation. 38 The optimal strategy (i.e., drug and dose) is unknown and depends on the site and etiology of bleeding which inform the short-term risk of rebleeding and its consequences. Although there is limited evidence to guide practice, prophylactic intensity anticoagulation (either oral or parenteral) can be considered in this setting once deemed safe to do after bleed cessation followed by titration to therapeutic intensity with close monitoring. For cases with unacceptably high bleeding risk (e.g., cerebral amyloid angiopathy), nonpharmacological alternatives like left atrial appendage occlusion for AF may be appropriate.

Patient/caregiver engagement is essential prior to restarting anticoagulation, especially when there is a substantial risk of rebleeding. Discussions should outline the expected benefit of restarting anticoagulation in terms of reducing thromboembolic risk, the potential harms of rebleeding, and education about clinical signs of bleeding.

### Gastrointestinal Bleeding

In a systematic review and meta-analysis of observational studies, individuals who resumed anticoagulation had a 70% lower risk of thrombosis (RR 0.30; 95% CI 0.13–0.68) but a 2-fold higher risk of rebleeding (RR 1.91; 95% CI 1.47–2.48). 89 Similarly, a population-based cohort study demonstrated almost 50% lower rate of thrombosis (HR 0.56; 95% CI 0.44–0.71) and a 2-fold higher rate of rebleeding (HR 2.02; 95% CI 1.69–2.40) among those who resumed anticoagulation compared with those who did not. 87 A recent multicenter retrospective cohort study of 948 patients hospitalized with GI bleeding (418 [44%] of whom were on a DOAC prior to the index bleeding event) showed that patients who resumed anticoagulation had a higher risk of clinically relevant bleeding (HR 1.55; 95% CI 1.08–2.22), lower risk of thromboembolism (HR 0.34; 95% CI 0.21–0.55), and lower risk of death (HR 0.50; 95% CI 0.36–0.68). Patients who underwent endoscopy were at lower risk of recurrent major bleeding (HR 0.69; 95% CI 0.39–0.94), whereas the timing of anticoagulation resumption (7, 14, or 21 days after bleeding) did not appear to influence the risk of rebleeding. 92 Prior studies showed that the highest bleeding rates were observed when warfarin was resumed within 7 days of GI bleeding compared with other time points. A mixed-methods study of physicians highlighted rebleeding risk and thrombosis risk as pivotal factors influencing decisions about resuming anticoagulation after gastrointestinal bleeding. 93 Surveyed physicians preferred resuming anticoagulation within 1 to 2 weeks for those at high thrombosis risk, while timing was more variable for those at low thrombosis risk, with many indicating resumption within 4 weeks or not at all.

### Intracranial Bleeding

The decision of whether and when to resume anticoagulation after intracranial bleeding is challenging and substantial equipoise exists. The risk of bleed recurrence must be balanced against the need for prevention of future ischemic events and is influenced by underlying etiology as well as individual patient risk factors. Traumatic intracranial bleeding is associated with an overall better prognosis and lower risk of recurrent bleeding after OAC resumption compared with spontaneous hemorrhagic stroke. 94 95 Limited existing evidence suggests that there may be overall net benefit in long-term all-cause mortality with anticoagulation reinitiation following intracranial bleeding, 91 96 97 98 99 100 101 but the ultimate decision is best made on an individual basis with input from specialists in neurology, neurosurgery, and stroke care. A 2017 systematic review and meta-analysis of 5306 patients with nontraumatic intracranial hemorrhage showed a lower risk of arterial thromboembolism (RR 0.34; 95% CI 0.25–0.45) and a comparable risk of rebleeding (RR 1.01; 95% CI 0.58–1.77) among those who resumed anticoagulation compared with those who did not. 91 However, in the 2017 systematic review and meta-analysis, all eight included studies were limited by retrospective observational design, almost all data evaluating only vitamin K antagonists, and likely prognostically important differences between groups at baseline (i.e., selection bias). Observational data from NASPAF-ICH suggest that the overall risk of rebleeding following reinitiation of anticoagulation after intracranial hemorrhage was nonsignificantly lower with non-vitamin K antagonists (NOACs) as compared with vitamin K antagonists (weighted risk ratio 0.72; 95% CI 0.38–1.38). 100 Published randomized trial evidence is also limited by trials that are likely underpowered to detect differences between groups due to small sample size. In the pilot noninferiority open-label SoSTART trial, starting OAC was not noninferior to avoiding OAC for the primary outcome of recurrent bleeding (adjusted HR 2.42; 95% CI 0.72–8.09) with nonstatistically significant (but possibly clinically significant) reduction in any symptomatic major vascular event (adjusted HR 0.51; 95% CI 0.26–1.03). 97 In the APACHE-AF trial, participants receiving apixaban had a nonstatistically significant (but possibly clinically significant) increase in the risk of intracerebral hemorrhage (adjusted HR 4.08; 95% CI 0.45–36.91) with a similar risk of major occlusive events (adjusted HR 1.05; 95% CI 0.48–2.31). 97 101 An individual participant data meta-analysis of 412 patients from four randomized trials (SoSTART, APACHE-AF, NASPAF-ICH, and a subgroup of patients in ELDERCARE-AF) showed that OAC therapy reduced the risk of major ischemic adverse cardiovascular outcomes (HR 0.27; 95% CI 0.13–0.56) compared with antiplatelet monotherapy or no OAC, but effects on the risk of any stroke, cardiovascular death, hemorrhagic events, or functional outcome were uncertain. The ongoing ENRICH-AF randomized trial (NCT03950076) is evaluating edoxaban versus usual care after intracerebral hemorrhage in patients with AF. Based on an early safety report showing a high risk of recurrent hemorrhagic stroke among patients receiving edoxaban with index lobar intracerebral hemorrhage or convexity subarachnoid hemorrhage, the trial data safety monitoring board recommended that edoxaban be discontinued in these patients and that no further patients with these bleed subtypes be enrolled. 102

The optimal timing of reinitiation following an index intracranial bleed is uncertain for patients for whom the decision is made to restart anticoagulation therapy. One study that examined warfarin restart following intracranial bleeding showed a net benefit (time point with the lowest rate of bleeding, thrombosis, and death following index bleed) at 7 to 8 weeks, while another study showed this time point to be at 10 to 30 weeks. 103 104 More recent data from a 2022 systematic review and meta-analysis showed a net benefit with anticoagulation restart as early as 2 to 4 weeks following index bleed. 105 A small retrospective observational study of mechanical heart valve patients with intracranial bleeding showed no difference in composite endpoint of symptomatic intracranial hematoma expansion, new intracranial bleed, incident acute ischemic stroke, or diagnosis of intracardiac thrombus among patients who restarted anticoagulation within 7 days (early restart group) versus waiting 7 to 30 days after intracranial bleed (late restart group) (HR 1.1; 95% CI 0.2–6.0). 106 Overall, there is significant variability in study findings and practice patterns regarding timing of anticoagulation reinitiation following intracranial bleeding. 107 Although there is no consensus, observational data show that, when indicated, antithrombotic therapy (including anticoagulation and antiplatelet therapy) is restarted most frequently within 3 to 6 months following intracranial hemorrhage. 98 Recent guidelines suggest resumption within 7 to 8 weeks is reasonable in patients for whom the benefit of treatment likely outweighs the harms and that alternative treatments should be considered as appropriate (e.g., left atrial appendage occlusion for patients with AF). 108

## Step 6: Prevent Future Bleeding

In keeping with principles of anticoagulant stewardship, long-term anticoagulant management necessitates regular follow-up, with a specific focus on mitigating bleeding risk by monitoring and addressing risk factors for bleeding. 109 Apart from measures to reduce the risk after specific bleeding events (such as administering proton pump inhibitors following peptic ulcer–related bleeds, or managing hypertension following intracerebral hemorrhage), general strategies to monitor/mitigate nonmodifiable risk factors and address modifiable ones can enhance safety. This encompasses ensuring the appropriateness of the drug and its dosage for the clinical indication and the patient's characteristics (e.g., age, weight) and existing health conditions (e.g., chronic kidney or liver disease) ( Table 1 ). Regular medication reviews can identify both pharmacodynamic interactions (e.g., interactions with antiplatelet therapies or nonsteroidal anti-inflammatory drugs [NSAIDs]) and pharmacokinetic drug–drug interactions (e.g., substances that induce or inhibit CYP3A4 and/or P-gp, the enzymes responsible for the metabolism of DOACs). 110 111 It is crucial to carefully reassess indications for concurrent antiplatelet therapy, a modifiable risk factor for bleeding. 112 113 Furthermore, a comprehensive review of herbal supplements is recommended to discontinue those that may interfere with platelet function. 114 115 Notable drug–drug interactions are summarized in Table 5 . Online tools can facilitate longitudinal assessments and documentation with quick reference checklists ( https://thrombosiscanada.ca/hcp/practice/clinical_tools?calc=vivomap329 ).

**Table 5:** Pharmacokinetic and pharmacodynamic interactions with DOACs

	Dabigatran	Edoxaban	Apixaban	Rivaroxaban
**Mechanism**	P-glycoprotein inhibition or induction		Combined cytochrome P450 3A4 and P-glycoprotein inhibition or induction	
**Drugs that increase DOAC concentration (contraindicated)**	Dronedarone glecaprevir/pibrentasvir, ketoconazole	Dronedarone, glecaprevir/pibrentasvir, ketoconazole	Ketoconazole, itraconazole, voriconazole, posaconazole	Ketoconazole, itraconazole, posaconazole, protease inhibitor (ritonavir), cobicistat, dronedarone
**Drugs that may increase DOAC concentration**	Amiodarone, cyclosporine, itraconazole, posaconazole, protease inhibitor (e.g., ritonavir), saquinavir, tacrolimus, tipranavir, quinidine, ticagrelor, verapamil	Amiodarone, cyclosporine, dronedarone, clarithromycin, erythromycin, itraconazole, posaconazole, ketoconazole, quinidine, verapamil HIV protease inhibitors (e.g., ritonavir)	Erythromycin, verapamil, diltiazem, naproxen, clarithromycin, protease inhibitor (e.g., ritonavir)	Erythromycin, clarithromycin, diltiazem, verapamil
**Drugs that decrease DOAC concentrations**	Rifampin, phenytoin, carbamazepine, phenobarbital	Rifampin, phenytoin, carbamazepine, phenobarbital	Rifampin, phenytoin, carbamazepine, phenobarbital	Rifampin, phenytoin, carbamazepine, phenobarbital
**Pharmacodynamic interactions that may increase bleed risk**	Antiplatelets (ASA and P2Y12 inhibitors), glucocorticoids, NSAIDs, prostacyclin analogs, SNRIs*, SSRIs*			
**Herbal supplements that decrease DOAC concentrations**	St John's Wort	St John's Wort	St John's Wort	St John's Wort
**Herbal supplements that may increase risk of bleeding**	Bilberry, bromelain, cat's claw, cinnamon, *Cordyceps* , danshen, dong quai, feverfew, flaxseed, garlic, ginger, *Ginkgo* , ginseng, melatonin, saw palmetto, turmeric, white willow			

Abbreviations: ASA, acetylsalicylic acid; DOAC, direct oral anticoagulant; NSAIDs, nonsteroidal anti-inflammatory drugs; SNRIs, serotonin-norepinephrine reuptake inhibitors; SSRIs, selective serotonin reuptake inhibitors.

## Case Resolution

### Case 1 (Intracranial Hemorrhage)

The initial goals of treatment for acute, spontaneous intracranial hemorrhage are to stabilize the patient's airway, breathing, and circulation, to avoid secondary neurologic insults (i.e., hypoxia, hypotension, hyperthermia, hypo- or hyperglycemia, hypercarbia), to assess the need for urgent surgery, to manage complications of bleeding (e.g., seizures), and to prevent hematoma expansion. Urgent neurosurgical consultation is required. This patient likely has clinically significant hemostatic impairment from rivaroxaban given that his last dose was taken 3 hours ago. A drug-specific anti-Xa assay can quantify the rivaroxaban drug level, but treatment should not be delayed pending results of this test. Rather, andexanet alfa (high dose regimen of 800 mg infusion followed by 960 mg infusion over 2 hours) or 4F-PCC 2000 IU (or up to 50 units/kg as per institutional guidelines) should be administered urgently depending on local institutional practice and drug availability. After hospital discharge, the risks and benefits of anticoagulation for secondary prevention of VTE should be reassessed. If this patient is eventually restarted on rivaroxaban, his blood pressure should be monitored closely in follow-up to reduce the risk of recurrent intracranial hemorrhage, and it may be reasonable to reduce the dose of rivaroxaban to 10 mg daily.

### Case 2 (Gastrointestinal Bleed)

This 68-year-old female has subacute gastrointestinal bleeding leading to anemia. Anticoagulation should be withheld, and supportive care initiated. Close inpatient observation is recommended, and idarucizumab should be administered for life-threatening bleeding. 116 TXA has not been shown to reduce the risk of death from acute gastrointestinal bleeding and appears to increase the risk of VTE when administered in high doses; therefore, its routine use is not recommended but may be considered on an individual basis. 43 Anticoagulation should be resumed with gastroenterology input and consideration of the etiology of bleeding, whether definitive hemostatic interventions were performed, and the risk and consequences of rebleeding (including whether future bleeding is likely to be amenable to definitive intervention). Patients treated with concomitant dabigatran and NSAIDs are at 2-fold higher risk of major bleeding compared with patients taking dabigatran alone. 117 Therefore, ibuprofen should be discontinued in favor of an analgesic that does not disrupt the gastrointestinal barrier or impair hemostasis (e.g., acetaminophen).

### Case 3 (Epistaxis)

This patient has nonsevere epistaxis which fulfills criteria for clinically relevant nonmajor bleeding as proposed by the ISTH for use in clinical research (bleeding that prompts face-to-face evaluation or medical intervention by a healthcare professional, or leads to hospitalization or an increased level of care but is not life- or limb-threatening). 40 Treatment may include topical vasoconstriction and/or nasal packing, and referral to otolaryngology is warranted for refractory or recurrent bleeding for definitive intervention. Depending on the degree of epistaxis and source control, anticoagulation with apixaban can either be continued or temporarily interrupted. If anticoagulation is interrupted, it should be resumed as soon as possible after the bleeding has stopped because the patient has a high risk of stroke or systemic embolism (AF; CHADS _2_ score of 5). Given the patient's elderly age of 87 years, the dose of apixaban should be reduced to 2.5 mg twice daily if his body weight is less than 60 kg and/or creatinine exceeds 133 umol/L. 51

## Conclusion

Severe bleeding complications associated with OAC use are associated with significant short-term and long-term adverse consequences including mortality and impaired quality of life. A protocolized, multidisciplinary approach to bleeding is needed to (i) rapidly identify patients with severe bleeding, (ii) optimize delivery of supportive measures, (iii) apply definitive treatments, and (iv) administer anticoagulant reversal or hemostatic therapies judiciously for patients most likely to benefit. Our guidance summarizes the latest evidence within a practical framework with key management considerations (i.e., rapid identification and treatment, considerations for restarting anticoagulation, engagement of patients/caregivers and multidisciplinary team, considerations for secondary prevention). With a corresponding algorithm that includes quick reference information for use at the point of care (e.g., coagulation tests and reversal/hemostatic therapies) and representative clinical cases, this guidance will support practicing clinicians to provide high-quality, multidisciplinary, holistic care for patients with severe bleeding complications related to anticoagulant therapies to improve outcomes and mitigate the potential harms of these effective treatments.
